# Ion Concentration-Dependent Ion Conduction Mechanism of a Voltage-Sensitive Potassium Channel

**DOI:** 10.1371/journal.pone.0056342

**Published:** 2013-02-13

**Authors:** Kota Kasahara, Matsuyuki Shirota, Kengo Kinoshita

**Affiliations:** 1 Department of Applied Information Sciences, Graduate School of Information Sciences, Tohoku University, Miyagi, Japan; 2 Tohoku Medical Megabank Organization, Tohoku University, Miyagi, Japan; 3 Institute of Development, Aging, and Cancer, Tohoku University, Miyagi, Japan; University of Zurich, Switzerland

## Abstract

Voltage-sensitive potassium ion channels are essential for life, but the molecular basis of their ion conduction is not well understood. In particular, the impact of ion concentration on ion conduction has not been fully studied. We performed several micro-second molecular dynamics simulations of the pore domain of the Kv1.2 potassium channel in KCl solution at four different ion concentrations, and scrutinized each of the conduction events, based on graphical representations of the simulation trajectories. As a result, we observed that the conduction mechanism switched with different ion concentrations: at high ion concentrations, potassium conduction occurred by Hodgkin and Keynes' knock-on mechanism, where the association of an incoming ion with the channel is tightly coupled with the dissociation of an outgoing ion, in a one-step manner. On the other hand, at low ion concentrations, ions mainly permeated by a two-step association/dissociation mechanism, in which the association and dissociation of ions were not coupled, and occurred in two distinct steps. We also found that this switch was triggered by the facilitated association of an ion from the intracellular side within the channel pore and by the delayed dissociation of the outermost ion, as the ion concentration increased.

## Introduction

Ion channels play essential roles in establishing accurate communications across plasma membranes. In particular, potassium (K^+^) ion channels are the most ubiquitously distributed proteins throughout the living world, conducting K^+^ ions with extreme selectivity, as compared to sodium ions [1]–[4]. In higher eukaryotes such as mammals, the K^+^ current through these channels is finely regulated, on the bases of the K^+^ gradient across the plasma membrane (∼5 mM and ∼100 mM outside and inside the cell, respectively) and the membrane voltage, and thus plays crucial roles in neuronal activities and the electrical conduction system of the heart. Illuminating the nature of this family of proteins is critically important to understand the molecular basis of living organisms.

The crystal structures of the ion channels have provided variable information to understand the molecular mechanisms of K^+^ conduction [5]–[10]. The ion conduction pore, which is formed by the interface of a homo-tetramer, can be roughly divided into two parts: the central cavity and the selectivity filter (SF). The SF, a quite narrow pore consisting of four residues (Thr-Val-Gly-Tyr) in each monomer, is the main player in the discrimination of K^+^ ions from other ions [11], [12]. From the crystal structures, the ion-binding sites in the SF were also identified, and it is considered to conduct K^+^ ions in a stepwise, single-file manner [5], [13].

To describe the dynamics of the K^+^ conduction processes by the SF, two possible different models have been proposed. In the one-step, “knock-on” model by Hodgkin and Keynes [14], K^+^ conductions are considered to occur by repeating a single step, where an incoming ion from the intracellular side enters the SF and the outermost ion in the SF is pushed out at the same time. On the other hand, in the two-step association/dissociation model (A/D model) by Nelson [15], [16], conductions are considered to occur in a two-step manner, called the association step and the dissociation step, where the dissociation of the ion at the outermost site is followed by the subsequent association of a new ion from the intracellular side. These mechanisms are difficult to observe directly by experimental techniques, and thus it has been hard to discriminate the models.

One of the powerful ways to gain insight into biological phenomena at the molecular level is the molecular dynamics (MD) approach, which can calculate the time courses of molecular systems, based on Newtonian mechanics. This method has been extensively applied to analyze the dynamics of ion conductions [17]–[22], but this approach is quite computationally demanding, because long simulation times are required for statistically relevant discussions of ion conductions.

One important milestone of MD approaches to ion conductions was presented by Jensen *et al.* [21]. They achieved microsecond-scale MD simulations with a voltage sensitive K^+^ channel from rat brain (Kv1.2), by using specialized hardware for MD simulations [23]. Their simulations revealed that K^+^ conductions by the Kv1.2 channel pore follow Hodgkin and Keynes's knock-on model, but their simulations were performed with a 600 mM KCl solution, where the ion conductance is considered to be saturated [24]. Their simulations were mainly focused on the voltage sensitivity of the ion channels, and thus their strategy to observe as many ion conductions as possible is quite reasonable. However, the extremely high ion concentration may cause some differences in the dynamic behaviors at the atomistic scale from lower ion concentration, e.g., physiological ion concentration [25].

In this study, we focused on the ion concentration effects on ion conduction, and presented distinct observations of K^+^ conductions by the same Kv1.2 channel as in Jensen's simulation. In our simulations, we used four systems with different ion concentrations, from 150 mM to 600 mM, with intervals of 150 mM. We performed long-term 3.0 µs MD simulations in total (1.0 µs for the 150 mM and 600 mM conditions, and 0.5 µs for the intermediate (300 mM and 450 mM) conditions). In addition, we analyzed the mechanisms of ion conduction from the trajectories by using an “ion-binding state graph”, where a set of binding positions of the K^+^ ions in the pore is treated as a node, and transitions among the states are treated as edges. As a result, we found that the ion conduction behavior gradually changes from the two-step A/D model at lower concentrations to the knock-on model at higher concentrations.

## Results and Discussion

### Conductance

**Figure 1:**
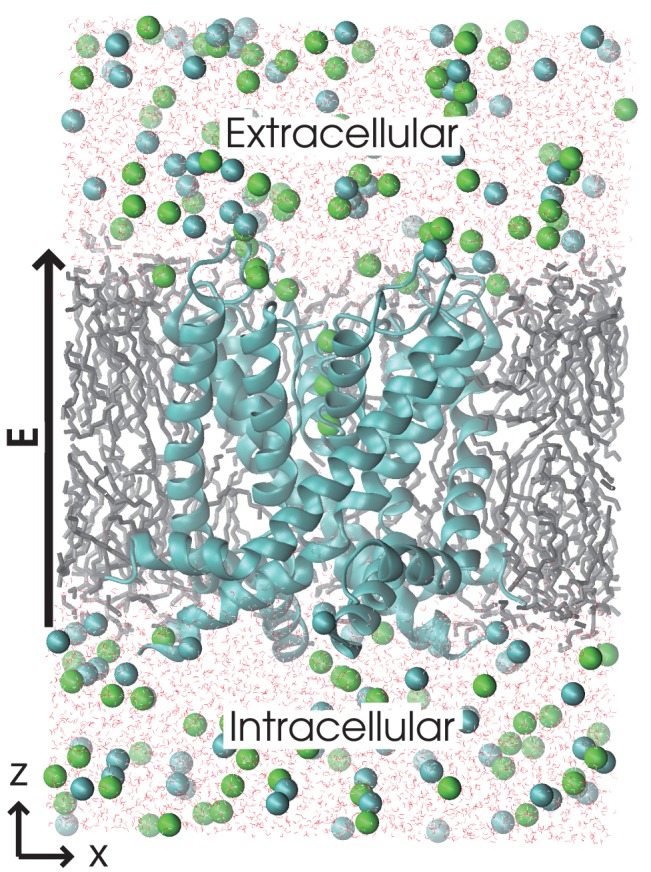
The simulation system at the 600 mM ion concentration. The system is composed of a homotetrameric Kv1.2 channel with paddle chimera (PDB-ID: 2r9r; shown as cyan ribbons), a POPE membrane (shown as gray sticks), and a KCl solution (waters, potassium ions, and chloride ions are depicted as red dots, green balls, and cyan balls, respectively). The electric field was applied toward the extracellular side (shown as the arrow labeled ‘E’).

We successfully observed repeating ion conduction events in the systems consisting of a homo-tetrameric pore domain of the Kv1.2 channel protein (which includes S5, S6, and the S4–S5 linker helices, obtained from PDB-ID: 2r9r), a POPE membrane, and KCl solution. We used four different concentrations of KCl: 150 mM, 300 mM, 450 mM, and 600 mM (Fig. 1; the numbers of waters and ions in each system are shown in Table S1), and each simulation time was 1.0 µs, 0.5 µs, 0.5 µs, and 1.0 µs, respectively. The system at the highest ion concentration (600 mM) was quite similar to that investigated by Jensen *et al.* [21]. During the simulations, the temperature and pressure were maintained at 310 K and 1.0 bar, respectively.

In these simulations, an electric field was applied to the entire system to as 920 mV of the membrane voltage, calculated by the following equation: *V = EL_z_* (*V* denotes the voltage, *E* denotes the electric field, and *L_z_* denotes the length of simulation box along z-axis). This condition is roughly consistent with the previous simulation by Jensen et al. referred as 123 mV of the membrane voltage in their paper [21], because they calculated the membrane voltage by the equation: *V = E* ***Δ*** *z* (***Δ*** *z* is the length of the SF). It should be noted that the membrane potential which a channel experiences under the electric field is equal to the potential drop not over the SF but over the entire system as demonstrated by Roux and colleagues by using the Poisson-Boltzmann equation [26] and all-atom MD simulations [27]. These theoretical studies are now widely accepted and Jensen *et al.* have changed the way to calculate the membrane voltage in their recent work [28].

In our simulations, 30, 31, 27, and 71 K^+^ ions permeated through the pore, at the four ion concentrations in ascending order, which were equivalent to 4.8 pA, 9.9 pA, 8.7 pA and 11 pA, respectively. The order of magnitude and the ratio of these values roughly agree with the experimental results with a Shaker channel, reported by Heginbotham and MacKinnon [24], and are comparable to the previous simulation by Jensen *et al.* [21]. There was an increasing trend in conductance with ion concentration, except for the lower conductance at 450 mM than that at 300 mM. This can be interpreted as trivial differences in the initial coordinates of each atom, which can produce such an exceptional outcome. Thus, even the long sampling over submicro-second may still be insufficient to estimate quantitatively the correct conductance, although the behaviors of the systems are qualitatively consistent.

As an example of some ion conduction events, a part of trajectory including some ion conduction processes at 150 mM ion concentration is shown in Movie S1 and Fig. S1.

### Binding site usage

**Figure 2:**
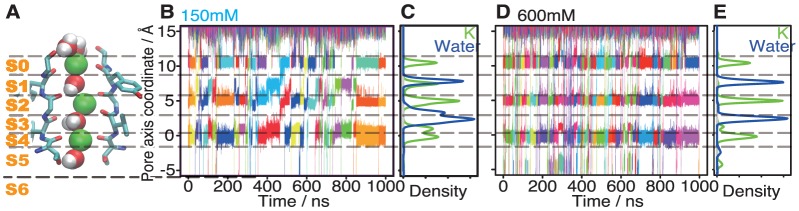
Trajectories of K^+^ ions. (A) The structure of the selectivity filter consisting of four residues (Thr-Val-Gly-Tyr) is shown with an additional Gly residue, to clarify the binding sites. Green, red, and white balls represent potassium, oxygen, and hydrogen atoms, respectively. (B, D) Trajectories of each K^+^ ion in the pore along the simulation time, for the simulations at 150 mM and 600 mM. The color of each line corresponds to the ID of each K^+^ ion. The pore axis is defined as the line between the centers of the four backbone carbonyl oxygen atoms of Thr-374 and those of Tyr-377. (C, E) Densities of K^+^ ion position (green line) and water molecule positions (blue line) at 150 mM (C) and at 600 mM (E) K^+^ ion concentrations.

To describe the ion conduction events through the channel pore schematically, we followed the definition of K^+^ binding sites based on carboxyl groups of the SF, as described by Jensen *et al.* [21]. With this definition, the pore has four internal ion binding sites, named S1–4, and three additional peripheral sites, called S0, S5, and the central cavity, denoted as S6 (Fig. 2A).

Firstly, the occupancy of the ion-binding sites was analyzed from the density maps of the ion locations along the pore axis. The results revealed the use of distinct sets of ion binding sites between the low and high ion concentrations (Fig. 2C and E). The density distribution at the highest ion concentration (600 mM; Fig. 2E) clearly shows that the conducted K^+^ ions bound exclusively to S0, S2, and S4, and the waters bound exclusively to the intervening sites: *i.e.*, S1 and S3. Note that water molecules did not always intervene the K^+^ ions; ratios of the number of conducted water molecules over those of K^+^ ions were 0.83, 0.84, 0.67, and 0.79, at the four ion concentrations in the ascending order, respectively. This result roughly agreed with an experiment reported by Ando *et al.* [29]. In the case of the lowest ion concentration (150 mM), the K^+^ ions were also retained in S1 and S3, in addition to the three sites used at the high ion concentration (Fig. 2C). This result qualitatively agreed with the electron density map around the selectivity filter of a KcsA channel at a 200 mM ion concentration, presented by Morais-Cabral *et al.* [30]. Furthermore, the peaks at S1 for K^+^ ions and waters overlapped well, which means that the K^+^ ions and waters can use the same binding sites, in contrast to the case of conduction at the high ion concentration. At the 300 mM and 450 mM concentrations, the systems show intermediate properties between 150 mM and 600 mM (Fig. S2). From these observations, we concluded that the binding sites usage gradually changes with the increasing ion concentration of the solution, which implies that changes in the ion concentration can cause differences in the ion conduction mechanisms.

We then monitored the number of K^+^ ions bound to the pore in each of the ion concentrations. At higher ion concentrations, larger number of ions were kept in the pore (Fig. S3). At 600 mM, the pore almost always retained three or four ions, because ions were continuously supplied from the intracellular fluid. In contrast, at 150 mM, the pore had only two ions during about half of the simulation time. This indicates that the release of the third ion from the pore in the three ion states occurred rapidly than the provision of a new ion at the lower ion concentrations.

### Graphical analysis of the ion-binding states

**Figure 3:**
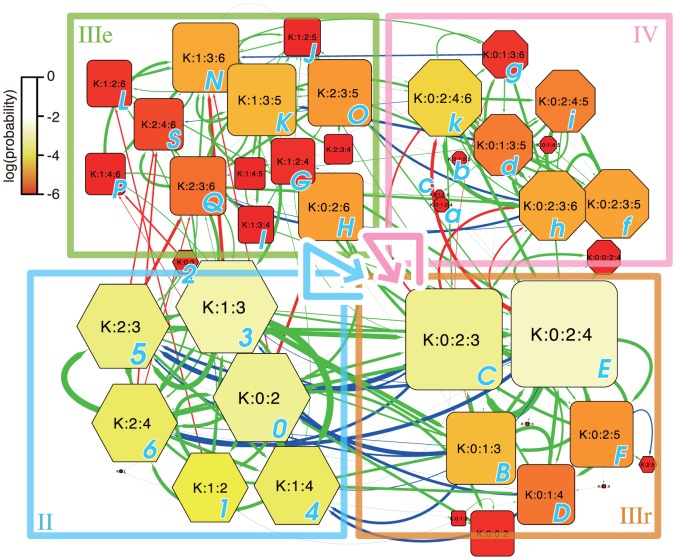
Ion-binding state graph. The ion-binding state graph for the simulation at 150 mM ion concentration. A node and an edge indicate the state of the binding ions in the pore, and the transition between two states, respectively. The size and color of each node mean log existence probability of the state during the simulation. The red edges mean that a new K^+^ ion is entering from the intracellular side, and the blue ones indicate that an ion in S0 is exiting to the extracellular side. The nodes are classified into the four groups: II, IIIr, IIIe, and IV, for the following discussion. The two major ways to conduct K^+^ are shown as cyan and pink bold arrows (see the main text for details). The graph was drawn with Cytoscape 2.8 [42].

The results described above imply that K^+^ ions are conducted in different manners between the high and low ion concentrations. To clarify the difference, the ion conduction processes were analyzed, using a graphical representation of the ion-binding states of the pore (Fig. 3 and Fig. S4, S5, and S6). The graph is referred to as “ion-binding state graph”, and its definition is described below.

The ion-binding state graphs were drawn according to the following steps: (i) the seven binding sites, S0–6, were identified by the pore axis coordinates (the thresholds of each site are shown as dashed lines in Fig. 2A; see Text S1 for details), (ii) the ion-binding states of the pore were defined based on the combinations of the binding sites containing ions; for example, when K^+^ ions bound to S0, S2, and S4, this state was denoted as “K∶0∶2∶4”, (iii) the ion-binding state in each snapshot was recorded, and a graph was drawn from the information about the frequencies of each state and that of the transitions between pairs of states. Here, a node means an ion-binding state, and a directed edge means a transition from one state to another. In addition, color and size of a node show the log existence probability of each state (more stable states are shown as brighter and larger). Each ion-binding state graph summarizes all of the ion-binding states and transitions observed during each simulation. Note that, in contrast to the schematic diagrams previously used [30]–[32], we explicitly considered the peripheral binding sites (denoted as S0, S5, and S6), in addition to the sites in the SF. We confirmed that the systems stationary behaved during the simulations, by comparing the existence probabilities of each ion-biding state between the first and last half of the simulation time (Fig. S7).

Figure 3 represents an ion-binding state graph for the 1.0 µs simulation at the 150 mM ion concentration. In this simulation, 42 states and 211 edges were identified. To interpret the ion-binding state graph, we will focus on two important events: the dissociation of the K^+^ ion in S0 to the extracellular side (blue arrows), and the association of a new K^+^ ion from the intracellular side (red arrows). K^+^ ion conductions occurred with repeating alternate transitions between these dissociation and association events.

**Figure 4:**
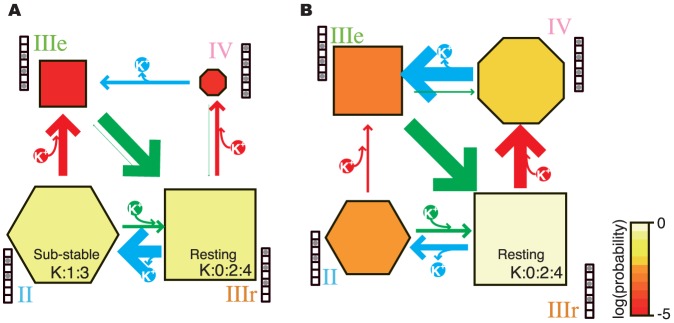
Simplified ion-binding state graphs. A simplified version of the ion-binding state graph shown in Fig. 3, according to the four groups of ion-binding states: II, IIIr, IIIe, and IV. Nodes labeled as II, IIIr, IIIe, and IV are correspond to the sets of ion-binding sites shown in the Fig. 3. (A) 150 mM. (B) 600 mM.

For the following discussion, the ion-binding states are classified into four groups, II, IIIr, IIIe, and IV, indicating the two-ion states, the three-ion states grouped with the “resting state”, the three-ion states as the “entrance” to the resting states (the meaning of it will be described later), and the four-ion states, respectively. According to this classification, Fig. 3 can be simplified as Fig. 4A, and it emphasizes the two significant events, *i.e.*, the association and the dissociation of an ion. The system at the IIIe state can easily transit to IIIr (many bold arrows from IIIe to IIIr, as shown in Fig. 3). Therefore, we can see the two types of cyclic pathways in this graph: (a) IIIr, II, IIIe, IIIr, and (b) IIIr, IV, IIIe, IIIr, shown in the order of the passed groups. This result indicated that the conduction of K^+^ ions can occur in one of two ways; that is, (a) via the two-ion states, and (b) via the four-ion states.

### Each ion conduction event on the graph

We also analyzed the time courses of the system on the graphs. In the simulation, the system began with the state K∶0∶2∶4. In following time steps, the system repeatedly moved to one of the successive nodes along an edge, or stayed at the same node, like a man on a Monopoly game board, and thus the trajectory of the system can be described as a path on the ion-binding state graph. Here, the trajectory was first divided into several short paths, corresponding to each conduction event. We then identified major ways to conduct K^+^ ions, by classifying each short path.

In the entire trajectory of the simulation at 150 mM, there were two stable states, K∶0∶2∶4 and K∶1∶3, with lifetimes of 38.4% and 22.2% of the simulation time, respectively, as consistent with previous simulations [17]. The state K∶0∶2∶4 can be regarded as the “resting state”. In the simulation, the system repeatedly made round trips, departing from the resting state and return to the same state. The time course of the simulation can be regarded as a set of round trips, or cyclic paths on the graph. In the simulations at the other ion concentrations, similar cyclic transition events were observed, with the resting state (or K∶0∶2∶4) existence probabilities of 57.0%, 74.2%, and 70.0%, at 300 mM, 450 mM and 600 mM KCl, respectively. Thus, these trajectories may be compared in terms of the variations in the cyclic paths on the ion-binding state graphs, due to the changes in the ion concentration.

There were 38 cyclic paths in the 1.0 µs simulation at the 150 mM ion concentration, which corresponded to 31 K^+^ ions passing through the pore from the intracellular side to the extracellular side (the inconsistency of these two values was due to some paths without ion conductions). On the other hand, there were 86 cycles with 71 K^+^ ion conductions in the simulation at 600 mM. Here, these 38 and 86 cyclic paths were compared and classified by hierarchical clustering, in order to discuss the underlying mechanisms of ion conduction at each ion concentration. Each path was represented as a sequence of characters, each of which corresponding to a state (for example, the one-letter code of the resting state, or K∶0∶2∶4, was assigned as ‘E’. See Fig. 3 for the other states). The 124 paths were converted into 124 sequences, and an all-against-all sequence alignment was performed by using the dynamic programming. The hierarchical clustering revealed two major clusters and some minor ones (Fig. S8; the clustering for all ion concentrations is shown in Fig. S9).

**Figure 5:**
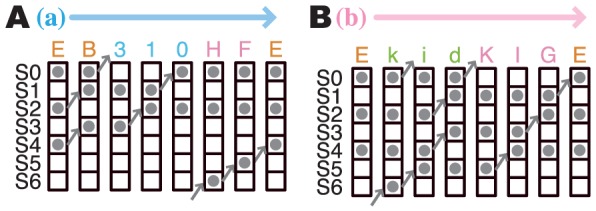
Schematic diagrams of ion conduction cycles and passage times of state transitions. Examples of schematic diagrams of ion conduction paths in each of the two major ways: via the two-ion states (A), and via the four-ion states (B). Each character shown means each ion-binding state, as shown in Fig. 3.

As an example of the first cluster, the typical path “EB310HFE” in Fig. 5A represents that (i) the system starts from the resting state (‘E’ or K∶0∶2∶4), (ii) the K^+^ ions at S2 and S4 move to the next sites (‘B’ or K∶0∶1∶3), (iii) the ion at S0 goes out to the extracellular side (‘3’ or K∶1∶3), (iv) the ions at S3 proceed to the next sites (‘1’ or K∶1∶2), (v) the S1 ion moves to S0 (‘0’ or K∶0∶2), (vi) a new K^+^ comes in from the extracellular side (‘H’ or K∶0∶2∶6), (vii) the new K^+^ ion moves to the next site by one (‘F’ or K∶0∶2∶5), and (viii) the system returns to the resting state (‘E’ or K∶0∶2∶4). This process can be considered to follow the A/D model, where the outermost K^+^ ion is released first (K∶1∶3), and then the system returns to the most stable state (K∶0∶2∶4) by receiving a new ion from the intracellular side.

In a similar way, the typical case of the other cluster was “EkidKIGE” (Fig. 5B), which can be interpreted as (i) the system starts from the resting state (K∶0∶2∶4), (ii) a new K^+^ comes in (‘k’ or K∶0∶2∶4∶6), (iii) the incoming ion goes up, and pushes the existing ones (‘i’ or K∶0∶2∶4∶5), (iv) the pushed ions push the neighboring K^+^ ions successively (‘d’ or K∶0∶1∶3∶5), (v) the outermost ion is pushed out (‘K’ or K∶1∶3∶5), (vi) the K^+^ in the innermost site pushes the upper K^+^ ion (‘I’ or K∶1∶3∶4 and ‘G’ or K∶1∶2∶4), and finally, (vii) the system returns to the resting state (‘E’ or K∶0∶2∶4). This path corresponds to the knock-on mechanism.

In short, these two major clusters correspond to the two ways described in the previous subsection: the way via the two-ion states, and that via the four-ion states. The classification of the conduction events confirmed that they were based on the two mechanisms, in which the former way via the two-ion states corresponds to the A/D model, and the other way, via the four-ion states, corresponds to Hodgkin and Keynes' knock-on model.

### Ion conduction mechanism switching by ion concentration changes

There were two major ion conduction paths at the lowest ion concentration as described above. Here, we examined effects of the ion concentration to the ion conduction mechanism. At the 150 mM ion concentration, the ratio of the cyclic paths via the four-ion states (knock-on mechanism) over those via the two-ion states (A/D mechanism) was 0.48 (10/21), which indicates that the A/D mechanism was preferred twice as much as the knock-on mechanism. This ratio gradually raised with increasing ion concentrations, as 0.67, 3.5, and 5.8, at 300 mM, 450 mM, and 600 mM, respectively (Table S2). In other words, an increase in the ion concentration switches the ion conduction mechanism from the A/D mechanism to the knock-on mechanism.

This alteration of the ion conduction mechanism may be interpreted from changes in the stability of each state. In the ion-binding state graphs (Fig. 3, S4, S5, and S6), the color and area of each node reflects log existence probability of each state (Fig. 3), which corresponds to the stability of a node. The simplified graphs for the simulations at 150 mM and 600 mM (Fig. 4A and 4B, respectively) clearly show that an increase in the ion concentration destabilizes two-ion states (II). When the pore in states II receives a K^+^ ion from the intracellular side, the state changed to IIIe and A/D conduction is occurred. Alternatively, if the pore is in states IIIr when a K^+^ ion comes, the state transits to IV and a K^+^ ion is conducted in the knock-on mechanism. Thus, the changing in the balance of the stabilities of states II and IIIr causes the alteration of the conduction mechanism.

**Figure 6:**
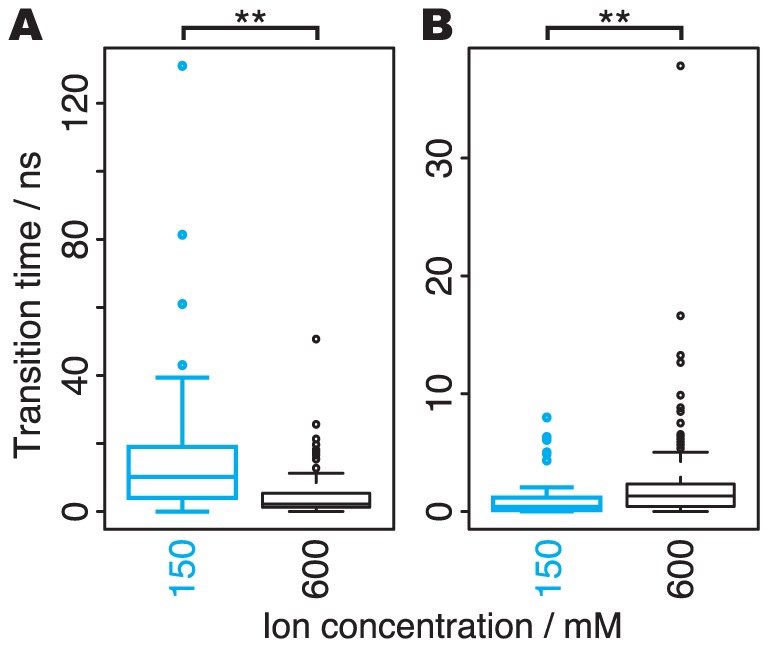
Times for ion association and dissociation events. Boxplots of times for the association (A) and dissociation (B) events, in the simulations at 150 mM (cyan) and 600 mM (black). Both of the events showed significant differences between the two ion concentrations; the P-values of Wilcoxon tests were 2.6e-5 (A) and 4.7e-3 (B). The increase in ion concentration facilitated the ion association, and delayed the ion dissociation.

The stabilities of states II, IIIr, IIIe, and IV will be determined by the balance between associations and dissociations of K^+^ ions, where frequent associations of K^+^ ions will lead the pore to the states with higher number of ions (IV), and *vice versa*. We analyzed times spent for each association and dissociation event (for example, in the conduction process shown in Fig. 5A, the time for the transitions from the first ‘E’ to ‘3’ was measured as a dissociation event, and that from ‘3’ to ‘H’ was an association event). As a result, the increase in ion concentration from 150 mM to 600 mM significantly accelerated the associations, and hinders the dissociations of ions (Fig. 6). These results indicate that the high ion concentration facilitated attacks of ions from the solution to the pore, and delayed releases of bound ions. Details of the transition times of each state are shown in Fig. S10 and discussed in the Text S1.

### Ions returning from the extracellular fluid

Some behaviors other than the two conduction mechanisms described above were also observed. Interestingly, after releasing the K^+^ ion in S0 from the three-ion state, another K^+^ ion occasionally entered from the extracellular side and bound to S0; *i.e.*, the system transited the following path, IIIr-II-IIIr. Our simulation showed that such back-running events frequently occurred during the outward conductions. For the resting state, *i.e.*, K∶0∶2∶4, a considerable part of its lifetime was with a back-ran K^+^ ion; the ratio of lifetime with a back-ran K^+^ ion over the entire lifetime of K∶0∶2∶4 were 64.3%, 45.2%, 26.1%, and 45.5%, for each ion concentration in ascending order. There was no clear trend between ion concentrations and back-ran K^+^ lifetimes. Because the back-running event can occur when a K^+^ ion attacks the pore in the two-ion states from the extracellular side, the two factors may be crucial for the occurrence of the back-running: the stability of two-ion states (denoted as II in Fig. 4), and the frequency of the attack of K^+^ ions. As an increase in ion concentration decreases the former and increases the latter, it is reasonable the fact that there was no clear trend in the ratio with the ion concentration.

The other minor ways were combinations of various events (see Table S2).

### Summary of the ion concentration-dependent ion conduction mechanism

Here, we will summarize the ion concentration-dependent ion conduction mechanism of a Kv1.2 channel. In the channel, the pore can bind two, three, or four ions in its seven binding sites, simultaneously. Basically, the pore adopts the most stable three-ion state, which is K^+^ ions in S0, S2, and S4. From this state, one of two types of events will occur: (a) one way pops the K^+^ ion from S0, and (b) the alternative one is the entrance of a new K^+^ ion from the intracellular side. In the former way (a), as the K^+^ ion in S0 is relatively unstable, it will eventually be released to the extracellular side (the transition from IIIr to II in Fig. 4). Then, the remaining two K^+^ ions move to S1 and S3, and the sub-stable two-ion state is adopted. Subsequently, the pore waits for a third ion, from either the intra- or extracellular side. When a K^+^ ion enters from the intracellular side, the state of the pore rapidly transits to the resting state (from IIIe to IIIr). This conduction event via the sub-stable two-ion state (K∶1∶3) corresponds to the A/D model. Alternatively, when a K^+^ ion enters from the extracellular side, the pore returns to the resting state (from II to IIIr). When we assume the actual situation (not the MD system with the periodic boundary condition), this event does not change the membrane potential (one K^+^ ion is released to the extracellular side, and another one comes from the same side). The latter event in the resting state (b) will occur if a K^+^ ion enters from the intracellular side before the dissociation of the K^+^ ion in S0 (this transition means from IIIr to IV). The pore assumes an unstable four-ion state, and the outermost K^+^ ion will be rapidly released to the extracellular side (from IV to IIIe). Subsequently, the pore smoothly returns to the resting state (K∶0∶2∶4; from IIIe to IIIr). This event corresponds to the knock-on conduction mechanism.

The preference for these events depends on the ion concentration. The barrier to the dissociation of the outermost K^+^ ion and that to the association of a new K^+^ ion from the intracellular side depend on the ion concentration (Fig. 6). An increase in the ion concentration facilitates the associations, and delays the dissociations. At the lowest ion concentration (150 mM), conductions by the A/D model were dominant (two-fold preferred over that by the knock-on model). In contrast, the knock-on model was six-fold preferred over the A/D model at the highest ion concentration (600 mM), in which ion conductance is saturated. The results of the simulations at the intermediate ion concentrations (300 mM and 450 mM) showed that the preference in the conduction mechanisms gradually changed with the increasing ion concentration. Our simulation revealed that the dependency of ion conductance on the ion concentrations is not only due to differences in the frequency of chances for attacking to the pore by ion diffusion but also by distinct conduction mechanisms.

## Methods

Simulation systems, consisting of the pore domain of the Kv1.2/Kv2.1 paddle chimera (PDB-ID: 2r9r [8]), a POPE bilayer, and a KCl solution were built with the Maestro and Desmond software [33]. The simulations for the four systems at 150 mM, 300 mM, 450 mM, and 600 mM ion concentrations were subsequently performed by GROMACS 4.5.3 [34], using Charmm27 forcefield [35] with CMAP correction [36]. TIP3P model was applied for water molecules. For potassium and chloride ions, the standard Charmm27 parameters were applied without any correction. An electric field was applied to imitate 920 mV of membrane potential to agree with Jensen's study [21], and the particle mesh Ewald method was applied for the long-range electrostatic potential. The bonds to hydrogen atoms were constrained by the LINCS algorithm [37]. The temperature of the systems was controlled at 310K by the Nosé-Hoover thermostat algorithm [38], [39], and the pressure was maintained at 1 bar by the Parrinello-Rahman method [40], [41]. For more details, see the Text S1.

## Supporting Information

Text S1 **Appendix.**
(PDF)

Table S1 **Numbers of water molecules and ions in each system.** Actual numbers of water molecules and ions consisting of each system are shown.
(PDF)

Table S2 **Summary of deduction events observed during the four simulations.** Summary of the cyclic paths based on node group II, IIIr, IIIe, and IV, defined in the manuscript. The values indicate the number of observations for each type of cyclic paths.
(PDF)

Movie S1 **A movie for ion conduction processes described in Fig. S1.**
(MPG)

Figure S1 **Snapshots of some ion conduction events at 150 mM of ion concentration.** Snapshots of the structure around the SF along the time course from 251,000 ps (panel A) to 290,680 ps (panel H) in the simulation under 150 mM of ion concentration. The trajectory including these snapshots is also shown as a movie, Movie S1. Sticks show the structure of SF residues; two of the four subunits are hidden for clarity. Balls shown in red and white indicate oxygen and hydrogen atoms; those in blue, orange, and purple are potassium ions. The strings started by “K:”, for example “K∶0∶2∶4” in the panel (A), indicate ion-binding states of the SF (see subsection “Graphical analysis of the ion-binding states” in Results/Discussion).
(EPS)

Figure S2 **Trajectories of K+ from the simulations at 300 mM and 450 mM ion concentrations.** See Fig. 2 in the main text.
(EPS)

Figure S3 **Ratio of the lifetimes of states with two, three, and four K^+^.** The ratio of the lifetimes of two-, three-, and four-states are shown as ‘II’, ‘III’, and ‘IV’, respectively.
(EPS)

Figure S4 **Ion-binding state graphs at 300 mM ion concentration.** The size and color of each node reflects the lifetime of each state in the simulation, and the width of each edge indicates the transition probability between the two nodes. A red edge means a new coming from the intracellular side, and a blue one means the release of the ion in the outermost site to the extracellular side. See also the legend of Fig. 3 in the main text.
(EPS)

Figure S5 **Ion-binding state graphs at 450 mM ion concentration.** See the legend of Fig. 3 in the main text.
(EPS)

Figure S6 **Ion-binding state graphs at 600 mM ion concentration.** See the legend of Fig. 3 in the main text.
(EPS)

Figure S7 **Lifetime of each state in the first and last half of a simulation.** Horizontal and vertical axes are the log lifetimes of each state during the first and last half of the simulation, respectively. Each point corresponds to each ion-binding state, as shown in Fig. 3. The high PCC, stands for Pearson correlation coefficient, showed high stationarity of these simulations at the 150 mM (A) and 600 mM (B).
(EPS)

Figure S8 **Cluster analysis of all of the cyclic paths in 150 mM and 600 mM.** Each sequence represents a cyclic path on the graph that begins at K∶0∶2∶4 and ends at the same state. A character was assigned to each state, and is shown as an orange character in each node, in which the first and last ‘E’ mean that the K∶0∶2∶4 state in the sequences was omitted. The sequences with the cyan arrow or marked with cyan asterisks are the conduction paths with the A/D model, and those with the pink arrow or pink asterisks are the path with the knock-on model. The sequences with orange asterisks represent other exceptional conduction events (see Table S2).
(EPS)

Figure S9 **All of the cyclic paths in the four simulations.** (A) Hierarchical clustering of the cyclic paths in the ion-binding state graphs for all four simulations. In addition to 38 sequences from the simulation at 150 mM (cyan) and 86 from that at 600 mM (black), 37 and 34 sequences from the simulations at 300 mM (green) and 450 mM (purple), respectively, were added. (B) Tables of the cyclic paths, ordered along the time course in each simulation. The color of the column “path in group” indicates the category of events: pink, cyan, and green mean a conduction by the knock-on, that by the A/D, and go and back-running events, respectively. The paths (a) and (b) corresponds the paths shown in Fig. 5A and B, respectively.
(EPS)

Figure S10 **Passage times of state transitions.** Boxplots displaying the time of each transition event between groups of the ion-binding states, in simulations at 150 mM (cyan) and 600 mM (black). Significant differences between the two ion concentrations were marked as ** and * (the P-value of Wilcoxon test ≤0.001 and ≤0.05, respectively). A) Transitions with association of an ion: from IIIr to IV, and from II to IIIe. B) Transitions with dissociation of an ion: from IIIr to II, and from IV to IIIe. C) the transition from IIIe to IIIr. The P-values were 3.6e-1 (A, left), 1.1e-4 (A, right), 8.2e-3 (B, left), 3.9e-2 (B, right), 9.9e-1 (C).
(EPS)
