# Supplementary material for: Ion Concentration-Dependent Ion Conduction Mechanism of a Voltage-Sensitive Potassium Channel
Source: PLoS One. 2013 Feb 13;8(2):e56342. doi: 10.1371/journal.pone.0056342 (PMC3572011; doi:10.1371/journal.pone.0056342)
Supplement: Text S1 — Appendix. (PDF) [file pone.0056342.s001.pdf]

Supporting Information Text S1: (Appendix)

Methods

System preparation

Our system was composed of the pore domain of the Kv1.2/Kv2.1 paddle chimera (consisting of the S5 and S6 helices and the S4-S5 linker; *i.e.*, residues 311-421; PDB-ID: 2r9r [1], a palmitoyl oleolyl phosphatidyl ethanolamine (POPE) bilayer, and a KCl solution. The system at 150 mM KCl was built with the System Builder module of Desmond [2], via the Maeastro software [3], and relaxed by gradually releasing the constraints and increasing the temperature. Using this structure, the systems with KCl solutions at 300 mM, 450 mM, and 600 mM were made by replacing appropriate numbers of water molecules with K^+ and Cl^- ions, respectively (Table S1). All of the systems were subjected to an equilibration run for 2 ns, and then we raised an electric field along the pore axis at a rate of 150 mV

per 1 ns up to 920 mV, by using the GROMACS software [4]. The system at the 150 mM KCl concentration contained 51,269 atoms in the periodic boundary cell, with 7.20 nm, 7.15 nm, and 9.78 nm for the X, Y, and Z axes, respectively, at the initial conditions.

Ion-binding state graph analysis

Here we describe the method for drawing the “ion-binding state graph”, which was used to discuss the ion conduction mechanism.

Permeations of K^+ ions through the SF were projected onto the one-dimensional movement along the pore axis coordinate, which is defined as the vector originating from the center of the four backbone oxygen atoms of Thr-374 and bound toward the center of the four backbone oxygen atoms of Tyr-377, since these residues comprise the extracellular and intracellular boundaries of the narrowest part of the SF, respectively. The trajectories of the simulations were sampled at 1 ps intervals, and the axis was recalculated for each snapshot.

The space in the SF was divided into seven binding sites, called S0 to S6. They were identified by the pore axis; coordinate with eight thresholds along the pore axis, namely, −15.0Å, −6.08 Å, −2.12 Å, 0.44 Å, 3.00 Å, 6.24 Å, 9.32 Å, and 12.93 Å from the origin, which were determined according to the minimum points in the density distributions (Fig. 2C). On the basis of the definition of binding sites, the "ion-binding state" was defined for each snapshot as the combination of the binding sites occupied by K^+ ions. For example, if three K^+ were bound at S0, S2, and S4 at a particular moment, then the state was designated as "K:0:2:4".

Finally, the population of each ion-binding state and the transitions between any pair of the states were counted during a simulation, and a directed graph, named an "ion-binding state graph", was generated, where each node is an ion-binding state and each edge corresponds to a transition between a pair of nodes (Fig. 3). The size of each node reflects the population of each state and each edge means the transitions between pairs of them. For expressing an ion conduction event as a string of states, each state was

labeled by a letter or number for the following analyses.

Dynamics of each ion conduction event

In order to inspect the ion conduction events, each sequence of transitions in the ion-binding state graph during the entire simulation was divided into several cyclic paths, departing from and returning to the most populated state, K:0:2:4. Here, the trajectories including the first and last three permeating K^+ ions were discarded, which means that we only used the paths containing the entire process, starting from the intracellular side to the extracellular side. To remove the trivial oscillation events between the neighboring states, we deleted the subcycles that did not account for ion conduction from each cyclic path. Then, each cyclic path was encoded by a sequence of labels for each state (Fig. 3).

For the sequences of cyclic paths, the all-against-all sequence alignment was performed by the dynamic programming. In the alignment, a score of 1.0 was added for each pair of aligned states when they had the same

number of binding ions. The alignment score between two states with different numbers of binding ions was defined as the cosine distance between two vectors, each composed of the transition probabilities from one of the two states to all of the states other than the two states. In addition, opening and extending a gap was allowed, with a penalty score of -1.0. Then, the sequences were clustered by the single-linkage method with alignment scores (Fig. S7, and S8).

Results and Discussion

Ion concentration dependence of each state transition event

To determine the steps influenced by the change in ion concentration, we evaluated the transition times between the states for low (150mM) and high (600mM) ion concentrations. We had expected that the attachment of an ion to the channel (transitions from II to IIIe, and from IIIr to IV) was concentration-dependent, while its release from the channel (transitions from IIIr to II and IV to IIIe) and the movement of ions in the channel

(transition from IIIe to IIIr) were not concentration dependent. We observed, however, that both the attachment and the release of an ion was affected by ion concentration as described below. First, an increase in ion concentration prominently accelerated the transition from II to IIIe states (Fig. S10, right), which can be interpreted due to an increase in the chances of an ion attack from the intracellular side to the pore by diffusion. In contrast, the transition time from IIIr to IV was not affected by the change in ion concentration (Fig. S10A, left). The reason why this ion-attaching transition was not delayed by the decrease in ion concentration would be due to the fact that the state IIIr is a bifurcating point, from which the system can move to the state II by the release of the K^+ ion in the S0 site. In other words, a channel cannot wait at the state IIIr for a long time, because it is likely to move to the state II in a relatively short time. On the other hand, an increase in ion concentration slowed the transition from IIIr to II states (Fig. S10B left), which, in contrast to our expectation, implies that ion releasing step is also concentration dependent, probably due to the interaction of the ions in the channel and

those in the bulk. It also slowed the other ion-releasing transition, IV to IIIe, although the difference in the transition time between the two ion concentrations was smaller than that in the transition from IIIr to II. Finally, the transition from IIIe to IIIr rapidly proceeded at both the 150 mM and 600 mM KCl concentrations (Fig. S10C). Thus, our simulation showed that an increase in ion concentration accelerated the attachment of ions to and delayed their release from the channel, indicating that the high ion concentration facilitated attacks of ions from the solvent to the pore, and blocked releases of bound ions by electrostatic repulsion.

Concerns in the simulation conditions

Although the molecular dynamics method has been well established, treatment of such a complex system, containing a membrane and protein with the electric field, is not straightforward. This section describes some concerns about our simulation conditions.

First, since our simulation system applied the periodic boundary

condition, there was no gradient of ion concentrations across the membrane in contrast to the physiological condition. This discrepancy may generate artifacts in the balance of association and dissociation events, because the association events are facilitated by the ion concentration of the intracellular side, and the other events is delayed by that of the extracellular side. The quantitative discussion of relationship between ion concentrations and the ratio of the A/D conductions over the knock-on ones may be difficult. Our simulation systems correspond to electrophysiological experiments for a single-cell measurement with the external electric field without ion concentration gradient across the membrane.

Second, what force field is the best for this case has not been clear. Where this study applied Charmm27 force field, several force fields and parameter sets have been proposed for proteins, lipids, waters and ions. Jensen *et al.* have reported difficulty to reproduce I-V property especially at low voltage area by Charmm27 [5]. This point should be tested in the future works.

References

1. Long SB, Tao X, Campbell EB, MacKinnon R (2007) Atomic structure of a voltage-dependent K^+ channel in a lipid membrane-like environment. Nature 450: 376–382. doi:10.1038/nature06265.

2. Bowers K, Chow E, Xu H, Dror R, Eastwood M, et al. (2006) Scalable Algorithms for Molecular Dynamics Simulations on Commodity Clusters IEEE. pp. 43–43. doi:10.1109/SC.2006.54.

3. Schrödinger - http://www.schrodinger.com/

4. Hess B, Kutzner C, van der Spoel D, Lindahl E (2008) GROMACS 4: Algorithms for Highly Efficient, Load-Balanced, and Scalable Molecular Simulation. J Chem Theory Comput 4: 435–447. doi:10.1021/ct700301q.

5. Jensen MO, Borhani DW, Lindorff-Larsen K, Maragakis P, Jogini V, et al. (2010) Principles of conduction and hydrophobic gating in K^+ channels. Proceedings of the National Academy of Sciences 107: 5833–5838. doi:10.1073/pnas.0911691107.
