# Supplementary material for: Ion Concentration-Dependent Ion Conduction Mechanism of a Voltage-Sensitive Potassium Channel
Source: PLoS One. 2013 Feb 13;8(2):e56342. doi: 10.1371/journal.pone.0056342 (PMC3572011; doi:10.1371/journal.pone.0056342)
Supplement: Table S1 — Numbers of water molecules and ions in each system. Actual numbers of water molecules and ions consisting of each system are shown. (PDF) [file pone.0056342.s002.pdf]

Supporting Information Table S1

Numbers of water molecules and ions in each system.

Ion concentration / mM	150	300	450	600
Water	9691	9645	9593	9535
K^+	35	58	84	113
Cl^-	27	50	76	105
