# Supplementary material for: Ion Concentration-Dependent Ion Conduction Mechanism of a Voltage-Sensitive Potassium Channel
Source: PLoS One. 2013 Feb 13;8(2):e56342. doi: 10.1371/journal.pone.0056342 (PMC3572011; doi:10.1371/journal.pone.0056342)
Supplement: Table S2 — Summary of deduction events observed during the four simulations. Summary of the cyclic paths based on node group II, IIIr, IIIe, and IV, defined in the manuscript. The values indicate the number of observations for each type of cyclic paths. (PDF) [file pone.0056342.s003.pdf]

Supporting Information Table S2

Ion concentration / mM	150	300	450	600
IIIr-IV-IIIe-IIIr (*)	9	12	19	55
IIIr-IV-IIIe-IV-IIIe-IIIr (**)	0	0	1	3
IIIr-IV-IIIe-IV-IIIe-IV-IIIe-IIIr (***)	0	0	0	1
IIIr-IV-IIIr (*)	1	0	0	0
IIIr-IV-IIIe-IIIr-IV-IIIe-IIIr (**)	0	0	1	0
IIIr-II-IIIr-IV-IIIe-IIIr (-*)	0	0	1	0
IIIr-II-IIIr-IV-IIIe-IIIr-IV-IIIe-IIIr (-**)	0	2	0	0
IIIr-II-IIIe-IIIr (†)	19	16	4	8
IIIr-II-IIIr-II-IIIe-IIIr (-†)	2	2	1	2
IIIr-II-IIIe-IV-IIIe-IIIr (†*)	0	1	0	2
IIIr-IV-IIIe-II-IIIe-IIIr (†*)	0	0	0	2
IIIr-II-IIIr (-)	5	3	7	10
IIIr-II-IIIr-II-IIIr (--)	0	0	0	2
IIIr-II-IIIr-IIIe-IIIr (-)	1	0	0	0
IIIr (‡)	1	1	0	1
Total	38	37	34	86

(*) Knock-on conduction, (†) A/D conduction, (-) back-running, (‡) an exceptional event; an ion comes into the cavity from the intracellular side and it returns to the fluid immediately.
